# The prognostic accuracy evaluation of SAPS 3, SOFA and APACHE II scores for mortality prediction in the surgical ICU: an external validation study and decision-making analysis

**DOI:** 10.1186/s13613-019-0488-9

**Published:** 2019-01-30

**Authors:** Antônio Luis Eiras Falcão, Alexandre Guimarães de Almeida Barros, Angela Alcântara Magnani Bezerra, Natália Lopes Ferreira, Claudinéia Muterle Logato, Filipa Pais Silva, Ana Beatriz Francioso Oliveira do Monte, Rodrigo Marques Tonella, Luciana Castilho de Figueiredo, Rui Moreno, Desanka Dragosavac, Nelson Adami Andreollo

**Affiliations:** 1 Intensive Care Unit, Discipline of Physiology and Surgical Metabology, Department of Surgery, Faculty of Medical Sciences, State University of Campinas (Unicamp), Tessália Viera de Camargo St. 126, University Town Zeferino Vaz, Campinas, São Paulo 13083-887 Brazil; 2 Unidade de Cuidados Intensivos Polivalente, Unidade de Cuidados Neurocríticos, Hospital de São José, Centro Hospitalar de Lisboa Central, Lisbon, Portugal

**Keywords:** Prognostic scores, Critical care, Surgical intensive care unit

## Abstract

**Background:**

The early postoperative period is critical for surgical patients. SOFA, SAPS 3 and APACHE II are prognostic scores widely used to predict mortality in ICU patients. This study aimed to evaluate these index tests for their prognostic accuracy for intra-ICU and in-hospital mortalities as target conditions in patients admitted to ICU after urgent or elective surgeries and to test whether they aid in decision-making. The process comprised the assessment of discrimination through analysis of the areas under the receiver operating characteristic curves and calibration of the prognostic models for the target conditions. After, the clinical relevance of applying them was evaluated through the measurement of the net benefit of their use in the clinical decision.

**Results:**

Index tests were found to discriminate regular for both target conditions with a poor calibration (C statistics—intra-ICU mortality AUROCs: APACHE II 0.808, SAPS 3 0.821 and SOFA 0.797/in-hospital mortality AUROCs: APACHE II 0.772, SAPS 3 0.790 and SOFA 0.742). Calibration assessment revealed a weak correlation between the observed and expected number of cases in several thresholds of risk, calculated by each model, for both tested outcomes. The net benefit analysis showed that all score’s aggregate value in the clinical decision when the calculated probabilities of death ranged between 10 and 40%.

**Conclusions:**

In this study, we observed that the tested ICU prognostic scores are fair tools for intra-ICU and in-hospital mortality prediction in a cohort of postoperative surgical patients. Also, they may have some potential to be used as ancillary data to support decision-making by physicians and families regarding the level of therapeutic investment and palliative care.

**Electronic supplementary material:**

The online version of this article (10.1186/s13613-019-0488-9) contains supplementary material, which is available to authorized users.

## Background

Surgical procedures continue to evolve, and patients with advanced age, frailty, and comorbidities are exposed to interventions with different levels of invasiveness, complexity, morbidity, and mortality—proposed classification systems grade complications from those procedures as simple symptomatic situations to conditions requiring surgical, endoscopic or radiological reintervention and life-threatening organ failure [1, 2]. Therefore, admission to ICU for postoperative recovery is common for surgical patients [1, 2]. Nevertheless, admission to ICU is associated with potentially harmful situations like invasive monitoring and painful procedures [3]. Thus, a precise evaluation of the initial clinical condition, the type of procedure, and the final operative status is necessary to inform patients and physicians about the risk of complications and poor outcomes and to aid tailoring proportional therapeutic efforts.

Among many proposed prediction scores, Sequential Organ Failure Assessment (SOFA), Simplified Acute Physiology Score 3 (SAPS 3) and Acute Physiology and Chronic Health Disease Classification System II (APACHE II) are prognostic models that use clinical and laboratory variables to predict in-hospital mortality [4–8]. APACHE II and SAPS 3 were derived from a cohort of general ICU patients, while a consensus panel proposed SOFA as an organ dysfunction measurement score. Their performance was extensively assessed in several populational subgroups including mixed surgical–medical patients, post-cardiovascular surgical patients, and oncologic patients with heterogeneous results [9–12]. Therefore, external validation remains essential to evaluate the accuracy of them in new population subgroups and in different settings of care over time.

Moreover, traditional statistical methods use metrics based on sensitivity and specificity to assess prediction model’s accuracy. However, the relationship between the measurement of accuracy and its clinical usefulness is a gray zone [13, 14]. The decision analysis approach is an alternative to evaluate the clinical significance of applying those models and provides information into the clinical consequences of using them [13, 14]. This strategy has been used to test for the net benefit of using SAPS II to end-of-life care decisions and to evaluate the net benefit of a new model based on CURB-65 and C-reactive protein to guide decision-making in ICU-admitted patients with success [15, 16].

This study aimed to validate and compare the performance of SOFA, SAPS 3 and APACHE II for intra-ICU and in-hospital mortalities as the target conditions in a cohort of mixed surgical patients admitted to ICU for postoperative recovery and to test whether they aid in the clinical decision-making.

## Methods

This study was a prospectively defined analysis of a registry-based data validation cohort, gathered from consecutively admitted patients to a surgical ICU of a tertiary university hospital in Brazil, from January 1, 2013, to December 31, 2016. Our electronic database is continuously fed with predefined clinical and laboratory information from every patient admitted to our surgical ICU. Patients were followed daily during their ICU stay and then tracked for their final hospital status as discharged or deceased. The target condition of interest was the death of any cause in ICU or hospital. Variables, coefficients, and equations used for the index tests (SOFA, APACHE II, and SAPS 3) calculations were based on original publications without any adjustment or updating and are available upon request [4–6, 8]. APACHE II, SAPS 3 and SOFA scores were calculated after the first day of ICU admission using data collected at the prespecified time frame. This study was a registry-based data analysis with outcomes and predictors available before the beginning of any form of statistical analysis. Therefore, the blindness of outcomes or predictors was not employed. We followed the standards for reporting diagnostic accuracy (STARD) statement and the transparent reporting of a multivariable prediction model for individual prognosis or diagnosis (TRIPOD) statement recommendations for validation studies (Additional file 1: Figure S1) [17, 18].

We did not perform any formal statistical method for sample size calculation and evaluated all patients available in our database for enrollment. However, considering that more than 100 events were observed for intra-ICU mortality and more than 250 events for in-hospital mortality, we believe that our sample size is satisfactory.

Patients eligibility criteria for study enrollment were age 18 or above and admission to surgical ICU for postoperative recovery of an elective or urgent surgical procedure. Patient data were excluded only if the target condition information was missing. Noteworthy, there were no patient’s exclusions after application of eligibility criteria. Our eligibility criteria were restrictive, allowing only surgical patients enrollment. These criteria contrast with original development cohorts of SAPS 3 and APACHE II. The SAPS 3 cohort included the first ICU admission of patients aged 16 or more and excluded data from patients lacking information about any admission or discharge variables. The APACHE II cohort consecutively included ICU-admitted patients for a medical or surgical reason and excluded patients that were missing any admission variable information or submitted to a coronary artery bypass graft surgery. These inclusion criteria are in contrast with our sample that enrolled patients submitted to any surgical procedure and enrolled those who had admission data missing. We handled missing values in predictor variables with multiple imputations. This procedure was performed with SPSS version 22 using a linear regression model. The variables included in the multiple imputation model were intra-ICU and in-hospital mortalities, age, sex, type of surgery, SAPS 3, APACHE II, and SOFA scores. Ten imputed datasets were created, and areas under the receiver operating characteristic curve had their sensitivities and specificities averaged to generate the final curve used in our results.

Our ICU provides a mixed model of care with full-time intensivists, nurses, assistants, respiratory therapists, dietitians, and attending physicians. A minimum standardized level of care was provided, consisting of a daily checklist called ABCD-preV (Additional file 2: Table S1) [19], in order to minimize therapeutic variations inside the population that could change the probability of the outcome and biased the results.

We evaluated the predictive performance of the index tests in a cohort of general surgical patients by estimating their discrimination and calibration. Discrimination reflects the capacity of a prediction model to differentiate between those who do and do not develop the defined target condition during the study period. For the measurement of discrimination, we used the concordance index (C-index) statistic through the calculation of the area under the receiver operating characteristic curve (AUROC) with intra-ICU or in-hospital mortality as the binary endpoints. A value of 0.5 for AUROC signifies chance and means that the predictor in analysis cannot distinguish between a positive or an adverse outcome while a value of 1 represents perfect discrimination. Discrimination was classified according to AUROC values as follows: 0.90–1 excellent, 0.80–0.90 good, 0.70–0.80 fair, 0.60–0.70 poor and 0.50–0.60 fail [20]. The DeLong method was used to compare whether differences between different models AUROC’s were statistically significant [21]. Calibration reflects how well intra-ICU and in-hospital mortalities predicted by each model agree with the observed outcomes. This relation was shown graphically by clustering patients in tenths of predicted risk according to each model and plotting the expected against the observed number of cases. A smoothed line was drawn over the entire predicted probability range to augment the observed correlation. A well-calibrated model predicts over a line slope around 45°. The calibration plot also indicates the magnitude and direction of the model’s miscalibrations. For statistical analysis of the model’s predictive performance, we employed the Hosmer–Lemeshow goodness-of-fit test [22]. In an adequate sample size, results with *p* values higher than 0.05 indicate a good agreement between the model’s predicted probabilities and observed outcome rates.

Median follow-up was calculated for intra-ICU and in-hospital periods according to the reverse Kaplan–Meier survival function that uses the event indicator reversed and censoring becomes the outcome of interest.

A decision curve analysis was developed to describe and compare the clinical utility of tested models. Logistic regression was used to convert the model’s calculated values into predicted probabilities of death. Patients were defined as high risk if their intra-ICU or in-hospital mortality probabilities were higher than the prognostic model set probability threshold. Net benefit for different threshold values of each model was calculated according to Vickers et al. and compared to the possible clinical strategy of considering that all patients were positive for the outcome and treated them all and that all patients were negative for the outcome and received no treatment [13, 14].

Statistical analyses were performed using MedCalc version 18 and SPSS version 22. Continuous variables were reported as a mean and standard deviation or median and interquartile ranges whether they follow a normal distribution or not. Categorical variables were presented as count and proportion. Univariate analysis was performed using appropriated tests for continuous and categorical variables to assess association with mortality. Relative risks for mortalities were calculated after adjustment for illness severity. This procedure was performed using a case-control matching strategy with severity scores (SOFA, SAPS 3, and APACHE II) as specific criteria. A two-tailed *p* value of less than 0.05 was considered statistically significant.

## Results

**Fig. 1:**
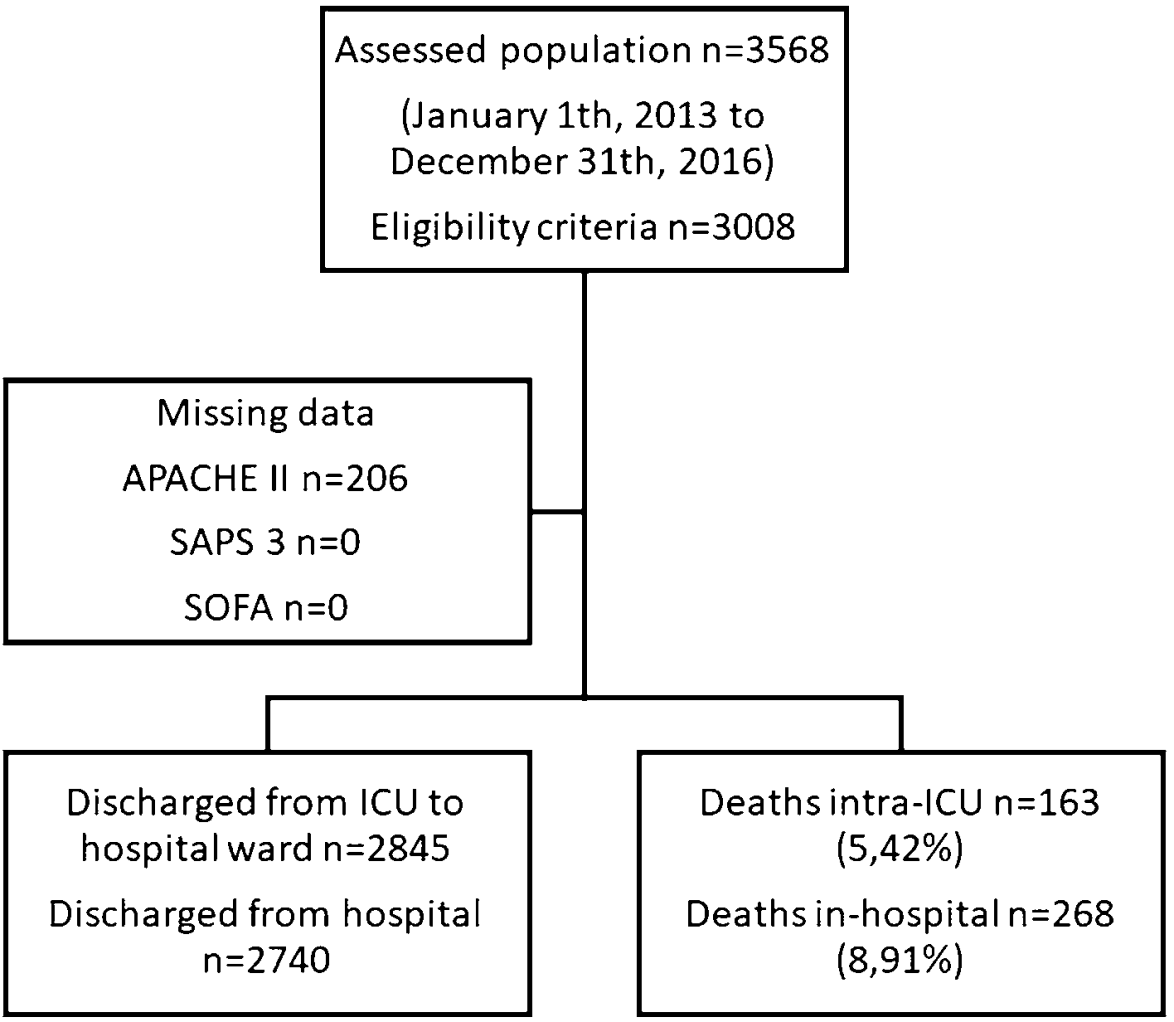
Participant flow diagram

We assessed an initial population of 3568 patients and polled out 3008 patients for further analysis according to our eligibility criteria (Fig. 1). The main reason for exclusion was ICU admission motivated by a medical reason not related to a surgical procedure. All patients assessed had their outcomes available, and no further exclusion was necessary. APACHE II, SAPS 3, and SOFA were calculated at appropriated timepoints and patients followed until they deceased or discharged from the hospital. APACHE II data were missing in 206 patients and had their values calculated using multiple imputations. Analyzed population demography and clinical features are summarized in Tables 1 and 2 and Additional file 3: Figure S2. In-hospital and intra-ICU mortality rates were 8.91% and 5.42%, respectively, during the evaluated period. Median follow-up period was 12 days for in-hospital length of stay and three days for intra-ICU length of stay. Mechanical ventilation was associated with the highest relative risk for ICU mortality [RR 3.97 (95% CI 1.59–9.95)].

**Table 1:** Patient’s baseline characteristics

	Total	Intra-ICU				In-hospital			
		Alive	Deaths	*p* value	Relative risk (95% CI)	Alive	Deaths	*p* value	Relative risk (95% CI)
Age median (IQR)	58 (47–67)	58 (47–67)	63 (53–70)	< 0.001*		57 (46–67)	63 (54.5–71)	< 0.001*	
Male sex count (%)	1798 (59.8)	1693	105	0.21**		1631	167	0.37**	
*The urgency of the surgical procedure count (%)*									
Urgent	220 (7.3)	170	50			152	68		
Elective	2788 (92.7)	2675	113			2588	200		
*Preexistent conditions count (%)*									
Arterial hypertension	1537 (51.1)	1452	85	0.72**		1394	143	0.40**	
Diabetes mellitus	634 (21.1)	604	30	0.39**		570	64	0.24**	
Alcohol use	371 (12.3)	347	24	0.34**		335	36	0.57**	
Tobacco use	1085 (36.1)	1029	56	0.64**		1001	84	0.09**	
Intra-ICU length of stay days median (IQR)	3 (2–5)	3 (2–5)	7 (3–15)	< 0.001*					
In-hospital length of stay days median (IQR)	12 (8–20)					11 (7–19)	17 (9–34.5)	< 0.001*	
*Severity Scores median (IQR)*									
SOFA	3 (2–6)	3 (2–6)	7 (5–9)	< 0.001*		3 (2–6)	6 (4–9)	< 0.001*	
APACHE II	12 (9–15)	11 (8–14)	17 (13–22)	< 0.001*		11 (8–14)	16 (13–20)	< 0.001*	
SAPS 3	36 (28–44)	36 (28–43)	52 (43–60)	< 0.001*		35 (28–43)	48 (41–58)	< 0.001*	
*Life support therapies*									
Mechanical ventilation count (%)	1491 (49.6)	1333	158	< 0.01**	3.97 (1.59–9.95)	1269	222	< 0.01**	1.44 (1.07–1.93)
Length of mechanical ventilation days median (IQR)	1 (1–2)	1 (1–1)	7 (2–12)	< 0.01*		1 (1–1)	5 (2–11)	< 0.01*	
Renal replacement therapy count (%)	143 (4.8)	93	50	< 0.01**	1.9 (1.42–2.53)	78	65	< 0.01**	1.78 (1.43–2.22)

*Mann–Whitney

**Chi-squared

**Table 2:** Type of surgery distribution across patients

Surgical specialties	Number of cases count (*n*)	Percent
*Head and neck surgery*		
Tumor	38	1.26
Others	14	0.47
*Cardiac surgery*		
Coronary artery bypass graft	339	11.27
Thoracic aortic aneurysm	89	2.96
Cardiac transplant	24	0.80
Valve replacement	189	6.28
Others	50	1.66
*Surgery of esophagus and abdomen*		
Liver	67	2.23
Liver transplant	141	4.69
Biliary tract	133	4.42
Esophagus and stomach	177	5.88
Colon, rectum, and anus	195	6.48
Others	4	0.13
*Neurosurgery*		
Aneurysm	105	3.49
Epilepsy	84	2.79
Tumor	317	10.54
Spine	109	3.62
Decompressive craniectomy	23	0.76
Ventriculostomy	23	0.76
Others	60	1.99
*Thoracic surgery*		
Tumor	70	2.33
Other	57	1.89
*Urology*		
Kidney transplant	123	4.09
Tumor	167	5.55
Others	48	1.60
*Vascular surgery*		
Abdominal aortic aneurysm	164	5.45
Endarterectomy	88	2.93
Others	95	3.16
Trauma, orthopedic, and ophthalmic surgeries	15	0.50
Total	3008	100

**Fig. 2:**
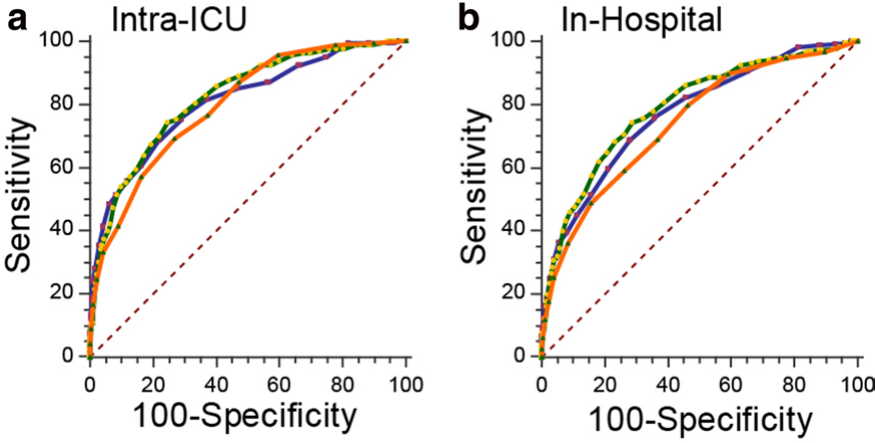
Pairwise comparison of the prediction model’s receiver operating characteristic (ROC) curves. ROC curves of different severity scores with intra-ICU (**a**) and in-hospital (**b**) mortality as the outcome. Green line—APACHE II; blue line—SAPS 3; orange line—SOFA

C-index statistics were calculated for each prognostic model with intra-ICU and in-hospital mortalities as dependent target conditions (Table 3). The following AUROCs were obtained with intra-ICU mortality as the outcome: APACHE II 0.808 (95% CI 0.794–0.822), SAPS 3 0.821 (95% CI 0.807–0.835), and SOFA 0.797 (95% CI 0.783–0.812). Considering in-hospital mortality, the following AUROCs were observed: APACHE II 0.772 (95% CI 0.757–0.787), SAPS 3 0.790 (95% CI 0.775–0.804), and SOFA 0.742 (95% CI 0.726–0.758). Pairwise comparison among prognostic models resulted in no significant difference between them, except for SAPS 3 and SOFA score AUROCs difference that could not be explained by chance when in-hospital mortality was the target condition (Table 4; Fig. 2).

**Table 3:** Severity score’s area under the receiver operating characteristic (AUROC) curves for hospital and ICU mortalities as outcomes

Severity score	AUROC—in-hospital mortality (95% CI)	AUROC—intra-ICU mortality (95% CI)
APACHE II	0.772 (0.757–0.787)	0.808 (0.794–0.822)
SAPS 3	0.790 (0.775–0.804)	0.821 (0.807–0.835)
SOFA	0.742 (0.726–0.758)	0.797 (0.783–0.812)

**Table 4:** Pairwise comparison of prediction scores AUROC curves

Severity score	Difference between AUROCs in-hospital mortality (95% CI)	*p* value	Difference between AUROCs intra-ICU mortality (95% CI)	*p* value
APACHE II versus SOFA	0.0296 (− 0.004 to 0.063)	0.0840	0.0109 (− 0.027 to 0.049)	0.5748
APACHE II versus SAPS 3	0.0177 (− 0.014 to 0.049)	0.2686	0.0130 (− 0.024 to 0.05)	0.4973
SAPS 3 versus SOFA	0.0474 (0.013–0.082)	0.0068	0.0263 (− 0.013 to 0.061)	0.2050

**Fig. 3:**
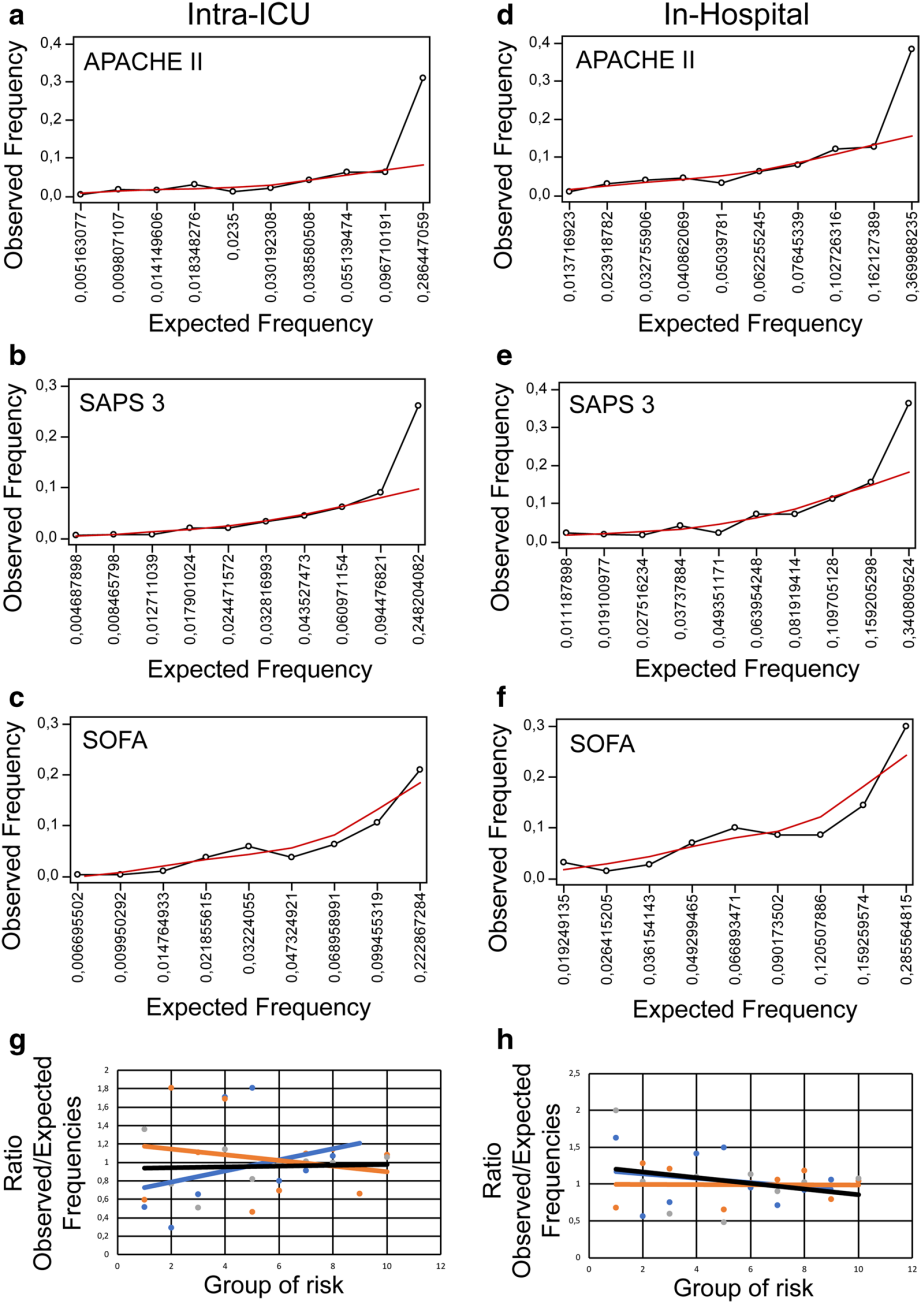
Prediction models calibration plots. **a**–**f** Groups covering the entire predicted intra-ICU (**a**–**c**) or in-hospital (**d**–**f**) mortality probabilities calculated by each severity score (on the *x*-axis) plotted against observed frequencies (on the *y*-axis) (Dots linked by the black line). A LOWESS line (red), spanning 75% of local values, was created for each dataset to clarify the relationship between assessed variables and to shed light on the direction and magnitude of model miscalibration across the probability range. **g**, **h** The ratios of observed over expected intra-ICU (**g**) or in-hospital (**h**) mortality probabilities, calculated by each prediction model (on the *y*-axis), were plotted against sequential clusters of risk (on the *x*-axis) to allow direct comparison between severity scores. Linear trend lines were created to aid in comparison. Orange line—APACHE II; black line—SAPS 3; blue line—SOFA

Next, patients were divided into approximately ten similar groups of risk defined by increasing order of estimated risk according to each prognostic model and expected, and observed deaths were calculated in each group. Calibration graphs were built plotting the expected and observed values for each group and goodness-of-fit tested with the Hosmer–Lemeshow statistics (Fig. 3; Table 5). Also, the ratios of observed and expected number of deaths in each risk group were plotted to show the overall fit of the tested models (Fig. 3). In summary, models had a poor calibration in extremities of risk, overestimating and underestimating intra-ICU and in-hospital mortality, respectively. Based on the Hosmer–Lemeshow goodness-of-fit test, APACHE II and SAPS 3 had *p* values above 0.05 while SOFA score showed a *p* value lower than 0.05 which indicates miscalibration for both outcomes.

**Table 5:** Prognostic model’s calibration values for hospital and intra-ICU mortalities as outcomes

Severity score	Hospital mortality	*p* value	intra-ICU mortality	*p* value
	Hosmer and Lemeshow test—Chi-squared (DF)		Hosmer and Lemeshow test—Chi-squared (DF)	
SOFA admission	18.04 (7)	0.0118	14.98 (7)	0.0362
SAPS 3	10.71 (8)	0.2189	2.02 (8)	0.9804
APACHE II	7.89 (8)	0.4441	13.35 (8)	0.1003

**Fig. 4:**
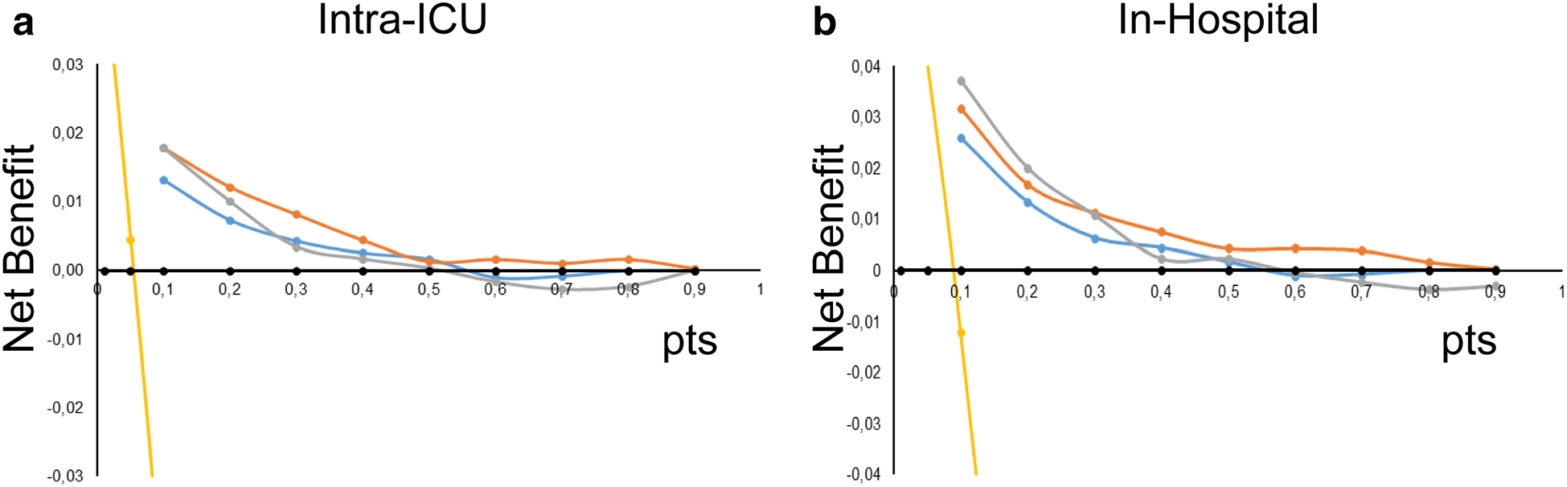
Prediction models decision curves. **a**, **b** The net benefits of using each prediction model (on the *y*-axis) plotted for different thresholds of the probability of intra-ICU (**a**) or in-hospital (**b**) deaths (on the *x*-axis). The net benefit was calculated according to the following formula: net benefit = [(true-positive count)/*n*] − [(false-positive count)/*n*] × [pt/(1 − pt)] where n is the total number of patients and pt the threshold probability. Two lines representing the net benefit associated with the strategy of assuming all patients survived (no false positives) (black line) and that all patients died (yellow line) was drawn for comparison. Orange line—APACHE II; blue line—SOFA; gray line—SAPS 3

Then, we calculated the intra-ICU and the in-hospital probability of death given by each prognostic model in ICU admission and plotted decision curves to determine how they aid in decision-making (Fig. 4). For both target conditions, the net benefit curves of the tested prognostic models were similar regardless of the selected threshold. Although SOFA, SAPS 3, and APACHE II showed diverse discrimination and calibration features, they showed a positive net benefit in the 10–40% range of death probability. Above or below this range, the net benefit of using them is no better than not treat any patient or treat them all, respectively.

## Discussion

In this external validation study, we sought to evaluate the performance of prognostic models to predict intra-ICU, and in-hospital mortalities in a cohort of surgical patients admitted in ICU for postoperative recovery and tested how it could help in decision-making. Multivariable prognostic models analyzed were employed identical to their original descriptions, without any adjustments in variables selection or weighting. SAPS 3 and APACHE II were initially developed to predict hospital mortality, while SOFA was initially proposed as a measurement of organic dysfunction and posteriorly validated for mortality prediction in different subgroups of patients [4, 5, 8, 23]. In development studies, SAPS 3 and APACHE II scores showed AUROCs of 0.825 and 0.863, respectively. In a recent review of prognostic scores performance in low and mid-income countries, discrimination of SAPS 3 and APACHE II evaluated through AUROCs ranged between 0.7 and 0.9 for intra-ICU and in-hospital mortalities as outcomes [24]. It is important to stress out that our sample was enrolled in a tertiary university hospital from a high-income region of Brazil and may have features different from low- and mid-income settings that may preclude extrapolation. To the best of our knowledge, none of the assessed prognostic models had their performance tested in a cohort exclusive of surgical patients from different specialties. Our data suggest fair to good discrimination of the tested models, with best results observed using SAPS 3 for prediction of both target conditions. APACHE II score was better calibrated for in-hospital mortality prediction than SAPS 3 and SOFA that trend to underestimate low-risk patient’s and overestimate high-risk patient’s probability of death. Scores prediction of intra-ICU mortality had a poor calibration with SAPS 3 fitting better among them.

In contrast to APACHE II and SAPS 3 that use features reflecting chronic conditions like the patient’s age to estimate risk, SOFA measures six organic variables reflecting mostly acute conditions. In this study, our sample was composed mainly of patients admitted to elective surgical procedures with their baseline conditions optimized. Perhaps SOFA performed poorly because of the lack of correlation between its variables and the target conditions in our setting. It is possible that recalibration of SOFA’s variables may improve its accuracy. Moreover, prognostic scores performance deteriorates over time and among different ICUs, especially calibration [25, 26]. Therefore, it is critical to external validate prognostic scores overtime and before their utilization in new ICUs.

The traditional evaluation of prognostic scores using discrimination and calibration measurements is not new and conventional, and cannot define whether is worth using a particular model as an ancillary tool for decision-making or which of them is superior in practice [13, 20]. We calculated the net benefit of tested models using different thresholds of the risk of death. Although death is a severe final event and false-negative and false-positive results limit the individual applicability of prognostic scores, the benefit of full therapeutic investment in certain patients admitted in ICU is unclear and may bring additional suffering and unnecessary resource utilization [13, 14]. Our data suggest that APACHE II, SAPS 3, and SOFA calculated in admission may add information to help physicians and patients in decision-making about therapeutic management and palliative care when the calculated predicted risk of death is between 10 and 40% with no score superior to others. Although redundant in extremes of illness severity, mortality of patients with low and intermediate levels of risk is difficult to predict and gathering data from prognostic models may improve decisions about therapeutic management [13, 14, 27]. It is important to stress out that there was no observed net benefit to patients with high levels of risk for both target conditions. Maybe the small sample size in this subgroup of patients was insufficient to create a detectable signal by the tested prognostic models.

This study has several limitations that must be stressed out. Our cohort was derived from a single-center population with inclusion and exclusion criteria that yielded significant differences in demographic and clinical features compared with original multicentric cohorts used for SAPS 3 and APACHE II development [4, 5, 8]. SAPS 3 and APACHE II cohorts were composed of mixed clinical and surgical cases, with almost half of patients being unplanned admitted in ICU, which contrasts with our sample that was composed exclusively of surgical patients admitted to ICU for postoperative recovery mainly of elective surgeries. Patients were also iller in original SAPS 3 and APACHE II development cohorts as illustrated by the number of organic dysfunctions which was higher than in our cohort. For instance, the median SOFA in SAPS 3 original development cohort was 9 with an interquartile range of 6–11, while our patients had a median SOFA of 3 with an interquartile range of 2–6 [5, 8]. Although the length of ICU and hospital stay, age, and comorbidities profile were similar among our patients and original SAPS 3 and APACHE II cohorts, comparison of intra-ICU and in-hospital mortality reveals differences in outcome rates [4, 5, 8]. SAPS 3 and APACHE II original cohorts exhibited a broad spectrum of intra-ICU and in-hospital mortalities, with rates ranging between 10 and 30%, while mortality rates observed in this study were both below 10%. This difference may be in part explained by the features described above in the composition of analyzed cohorts, but also from selection and information bias, which are intrinsic to observational studies [18]. Also, it must be pointed out that the time difference between each cohort assembly creates a variance in features like therapeutic options available at the time that have a direct impact on analyzed outcomes. SAPS 3 database was built from data of patients admitted in ICUs of multiple countries from October to December 2002, while APACHE II database recruited patients between 1979 and 1982 in multiple ICUs from the USA [4, 5, 8]. It is in contrast with our database which collected data from patients admitted in one hospital ICU from 2013 to 2016. Differences in frequency of tested outcomes are an important feature that may impact the generalizability of results and conclusions of external validation studies. Comparison of the observed in-hospital mortality rate in this study with those found in comparable cohorts showed similar frequencies [28–30]. Datasets from these studies were derived from elective and non-elective surgical patients in the postoperative period admitted in ICUs of European hospitals with similar features to the tertiary setting where our data were derived [28–30]. Correlation of our mortality frequencies with data from other Brazilian ICUs revealed similar in-hospital mortality although cohorts compositions were different [24, 31]. Another limitation was the small size of our cohort, especially in the high-risk subgroup of patients. This fact may account for part of the reasonable accuracy and poor calibration observed for the tested scores and the absence of net benefit to this subgroup of patients in decision-making.

## Conclusions

In conclusion, this study assessed the performance of widely used prognostic scores for death prediction of surgical patients admitted in ICU for postoperative recovery. Observed results suggested that APACHE II, SAPS 3, and SOFA have regular discrimination features and poor calibration. Other studies showed similar results in different population subgroups, none using a cohort with characteristics of ours. Currently, prognostic scores are used for benchmarking, comparisons between ICUs performance and standardization of excellence. As previously suggested by others, our data support the fact that adopting those prognostic scores without further local external validation and adjustment may be misleading [25, 26].

Another point to be stressed out is that although the tested prognostic scores have a net benefit in death prediction of the low and intermediate level of risk surgical patients admitted in ICU, their performance was deficient when applied in the high level of risk patients which is the subgroup most susceptible to the futility of care. Therefore, before being ascribed as ancillary tools to aid in decision-making, improvements in the net benefit features generated using the tested prognostic models, especially in extremes of illness severity, must be sought. Noteworthy, no prognostic model should be used isolated to guide decision-making or replace clinical judgment. Further studies are needed to define the exact role the tested prognostic models may have as part of the decision-making process in ICU.

## Additional files

**Additional file 1: Figure S1.** STARD 2015 Checklist: Prediction Model Validation.

**Additional file 2: Table S1.** ABCD-preV checklist.

**Additional file 3: Figure S2.** Prediction scores distribution frequency. A–F—Patients distribution across severity scores values with intra-ICU (A, C and E) and in-hospital (B, D and F) mortality as outcomes. Blue bars represent survivors and green bars non-survivors.

## Abbreviations

SOFA: Sequential Organ Failure Assessment
SAPS 3: Simplified Acute Physiology Score 3
APACHE II: Acute Physiology and Chronic Health Disease Classification System II
AUROC: area under the receiver operating characteristic curve
ICU: intensive care unit
