# Supplementary material for: The prognostic accuracy evaluation of SAPS 3, SOFA and APACHE II scores for mortality prediction in the surgical ICU: an external validation study and decision-making analysis
Source: Ann Intensive Care. 2019 Jan 30;9:18. doi: 10.1186/s13613-019-0488-9 (PMC6353976; doi:10.1186/s13613-019-0488-9)
Supplement: Supplementary file 2 — Additional file 2: Table S1. ABCD-preV checklist. [file 13613_2019_488_MOESM2_ESM.docx]

**Additional file 2: Table S1 – ABCD-preV checklist**

| **A – Alarms – Alarms parameterization including Calibrating (‘zeroing’) of pressures**  **B- Balance and Bundles – Fluid Balance and prophylactic actions for Ventilator-Associated Pneumonia (e.g., elevation of the head of the bed to 30-45 degrees, daily sedation suspension, and assessment of readiness to extubate), Deep Vein Thrombosis and Stress Ulcer**  **C – Checklist – Prescription Checklist including FAST-HUG and drug corrections for organ failures**  **D – Delirium – Delirium’s prevention including evaluation for daily awakening**  **PreV – Prevention/Protective mechanical ventilation strategy and surveillance for patient-ventilator asynchronies** |
| --- |
