# Supplementary figures and images for: The prognostic accuracy evaluation of SAPS 3, SOFA and APACHE II scores for mortality prediction in the surgical ICU: an external validation study and decision-making analysis
Source: Ann Intensive Care. 2019 Jan 30;9:18. doi: 10.1186/s13613-019-0488-9 (PMC6353976; doi:10.1186/s13613-019-0488-9)

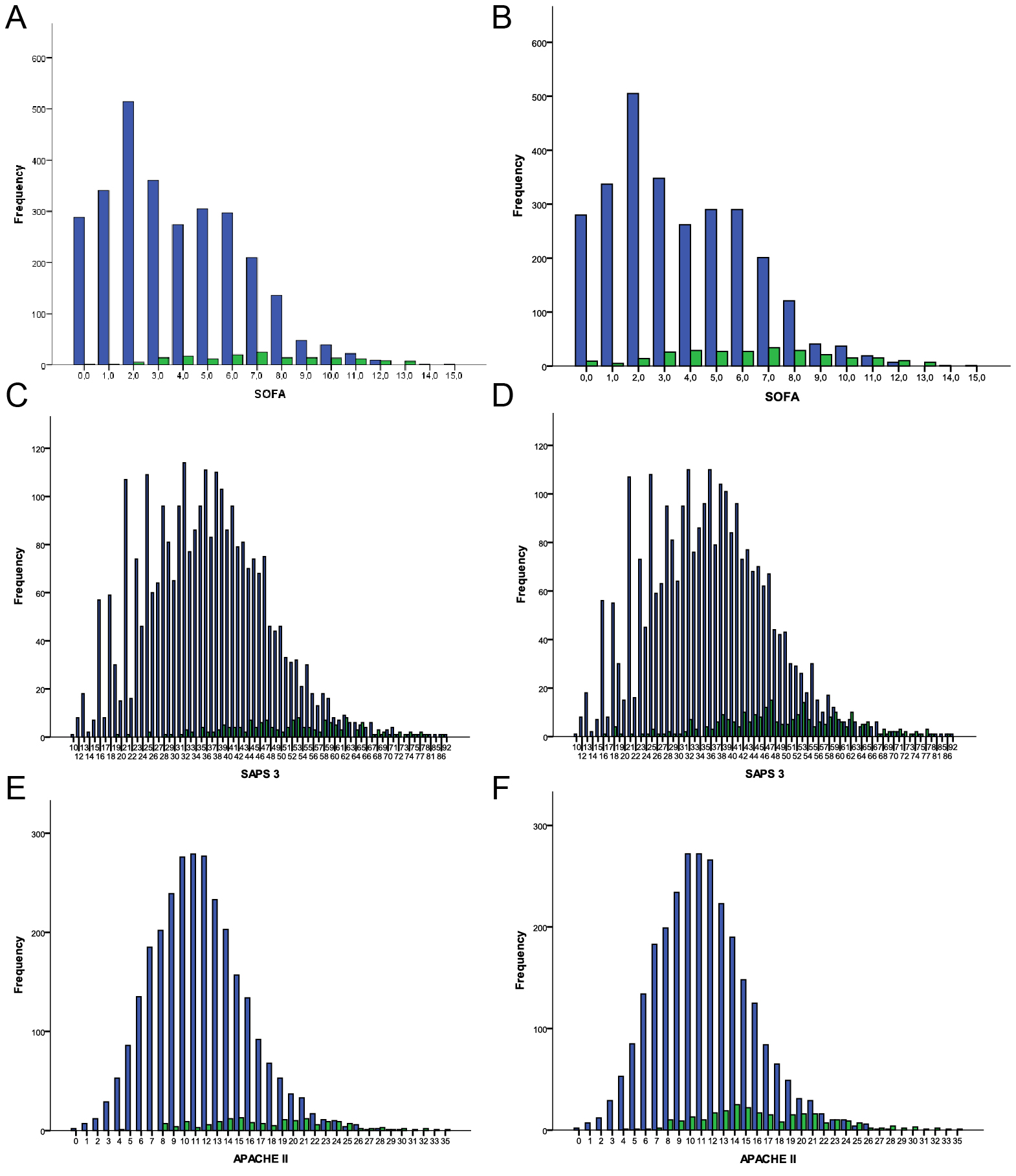

Supplement: Supplementary file 3 — Additional file 3: Figure S2. Prediction scores distribution frequency. A–F—Patients distribution across severity scores values with intra-ICU (A, C and E) and in-hospital (B, D and F) mortality as outcomes. Blue bars represent survivors and green bars non-survivors. [file 13613_2019_488_MOESM3_ESM.jpg]
